# Anti-GD2-IRDye800CW as a targeted probe for fluorescence-guided surgery in neuroblastoma

**DOI:** 10.1038/s41598-020-74464-4

**Published:** 2020-10-19

**Authors:** Lianne M. Wellens, Marion M. Deken, Cornelis F. M. Sier, Hannah R. Johnson, Fàtima de la Jara Ortiz, Shadhvi S. Bhairosingh, Ruben D. Houvast, Waleed M. Kholosy, Victor M. Baart, Annique M. M. J. Pieters, Ronald R. de Krijger, Jan J. Molenaar, Ellen J. Wehrens, Johanna F. Dekkers, Marc H. W. A. Wijnen, Alexander L. Vahrmeijer, Anne C. Rios

**Affiliations:** 1 Princess Máxima Center for Pediatric Oncology, Heidelberglaan 25, 3584 CS Utrecht, The Netherlands; 2 Oncode Institute, Hubrecht Institute – KNAW, Utrecht, The Netherlands; 3 Cancer Genomics Center, Utrecht, The Netherlands; 4 Department of Surgery, Leiden University Medical Center, Leiden, The Netherlands; 5 Department of Surgery, Maasstad Hospital, Rotterdam, The Netherlands

**Keywords:** Cancer models, Paediatric cancer, Translational research, Surgical oncology

## Abstract

Neuroblastoma resection represents a major challenge in pediatric surgery, because of the high risk of complications. Fluorescence-guided surgery (FGS) could lower this risk by facilitating discrimination of tumor from normal tissue and is gaining momentum in adult oncology. Here, we provide the first molecular-targeted fluorescent agent for FGS in pediatric oncology, by developing and preclinically evaluating a GD2-specific tracer consisting of the immunotherapeutic antibody dinutuximab-beta, recently approved for neuroblastoma treatment, conjugated to near-infrared (NIR) fluorescent dye IRDye800CW. We demonstrated specific binding of anti-GD2-IRDye800CW to human neuroblastoma cells in vitro and in vivo using xenograft mouse models. Furthermore, we defined an optimal dose of 1 nmol, an imaging time window of 4 days after administration and show that neoadjuvant treatment with anti-GD2 immunotherapy does not interfere with fluorescence imaging. Importantly, as we observed universal, yet heterogeneous expression of GD2 on neuroblastoma tissue of a wide range of patients, we implemented a xenograft model of patient-derived neuroblastoma organoids with differential GD2 expression and show that even low GD2 expressing tumors still provide an adequate real-time fluorescence signal. Hence, the imaging advancement presented in this study offers an opportunity for improving surgery and potentially survival of a broad group of children with neuroblastoma.

## Introduction

Neuroblastoma (NB) is the most common extracranial solid tumor occurring in children^1^. Over 75% of patients are categorized as high-risk with a poor overall 5-year survival of less than 50%, despite intensive treatment with high-dose chemotherapy followed by surgery, autologous stem cell transplantation, radiotherapy and immunotherapy^2,3^. Resection of high-risk NB is associated with a risk for serious surgical complications, since the tumor often encases major blood vessels leading to severe hemorrhage and/or unplanned organ damage^4,5^. Discriminating cancerous tissue from healthy tissue is challenging, but of great importance to achieve optimal tumor resection, while preserving healthy tissue, which can thereby increase survival^6,7^. Fluorescence-guided surgery (FGS) is a novel intra-operative imaging technique that empowers surgeons to visualize tumor tissue in real time using exogenous fluorescent agents. Most of the FDA-approved FGS probes are targeted against generic tumor markers^8^, with fluorescent 5-aminolevulinic acid (5-ALA) widely used for FGS on pediatric brain tumors^9^. However, recently, there has been a shift in this field towards the development of targeted probes that specifically bind surface markers on cancerous cells for molecular imaging^10^. Although the first cancer-targeted fluorescent agents have been evaluated in clinical trials for adults^11^, no specific probes have been developed so far for pediatric patients. GD2 is a disialoganglioside present on peripheral nerves and known to be overexpressed on most tumors from neuroectodermal origin, including NB^12,13^. GD2 represents a clinically relevant target, as patients with remnant NB are currently successfully treated with anti-GD2 immunotherapy after surgery^14,15^. Here, we conjugated this clinical grade antibody to the near-infrared (NIR) fluorophore IRDye800CW to explore its potential as a FGS probe for NB in preclinical settings.

## Results

### Anti-GD2-IRDye800CW specifically labels KCNR cells in vitro

**Figure 1:**
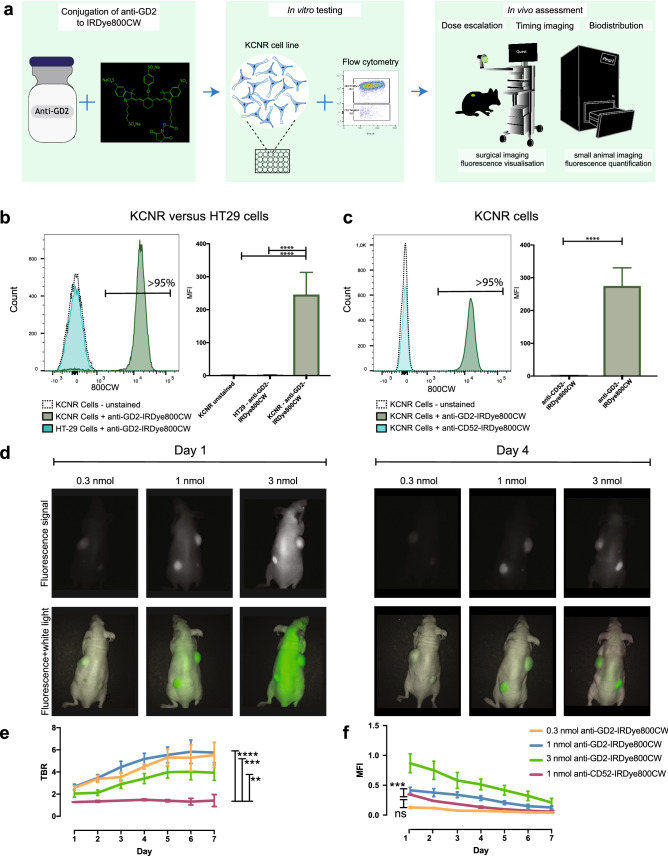
Dose escalating study in subcutaneous KCNR-derived tumor model reveals 1 nmol as the optimal dose and efficient real-time visualization of NB after 4 days. (**a**) Schematic overview of the preclinical evaluation pipeline. Anti-GD2 chimeric monoclonal antibody was conjugated to IRDye800CW (left panel) and evaluated in vitro on the NB KCNR cell line by flow cytometry (middle panel). In vivo validation was performed in NB cell line derived xenograft mouse models using the Pearl Trilogy Small Animal imaging system and Quest Spectrum imaging system (right panel). (**b**) Representative histogram (left panel) and accumulative data (right panel) of anti-GD2-IRDye800CW staining by flow cytometry on KCNR and HT-29 cells, compared to unstained cells. **c)** Representative histogram (left panel) and accumulative data (right panel) of anti-GD2-IRDye800CW staining on KCNR cells compared to CD52-IRDye-800CW staining. (**b**, **c**) n = 3 independent experimental repeats. Graphs depict mean + SEM, ****p < 0.0001. (**d**) Representative images using the surgical imaging device of mice bearing subcutaneous human KCNR-derived tumors, acquired 1 day (left panel) and 4 days (right panel) after administration of 3 ascending doses of anti-GD2-IRDye800CW. (**e**) TBR for 7 consecutive days of mice receiving different doses of anti-GD2-IRDye800CW or 1 nmol anti-CD52-IRDye800W as a negative control. Mean ± SEM. ****p < 0.0001; ***p = 0.0006 and **p = 0.0018 for comparison of 1, 0.5, and 3 nmol dose anti-GD2-IRDye800CW, respectively, to 1 nmol dose anti-CD52-IRDye800W. (**f**) MFI for all concentrations in arbitrary units. Mean ± SEM. ***p < 0.005 for comparison of 1 nmol dose anti-GD2-IRDye800CW to 0.3 nmol dose and non-significant (ns) for comparison of 0.3 nmol anti-GD2-IRDye800CW to control 1 nmol anti-CD52-IRDye800CW (*p* = 0.08). (**d**–**f**) n = 2 independent experiments with 3 to 4 mice per group.

Specific binding of anti-GD2-IRDye800CW was evaluated on the widely used patient-derived NB cell line; SMS-KCNR (KCNR)^16^ using an established evaluation pipeline^17^ that includes flow cytometry in vitro and fluorescence molecular imaging in vivo (Fig. 1a). Specific binding of anti-GD2-IRDye800CW to NB cells was observed by flow cytometry, with > 95% of cells staining positive, while a negative control colorectal cancer cell line; HT-29, showed no staining, similar to unstained cells (Fig. 1b). In addition, control anti-CD52-IRDye800CW, specific for CD52 (CAMPATH-1) present on the surface of mature lymphocytes, did not label KCNR cells (Fig. 1c). Overall, this demonstrates specific binding of anti-GD2-IRDye800CW to GD2 expressing KCNR cells in vitro.

### Effective KCNR-derived tumor detection in vivo and identification of optimal dose and imaging time window

We next addressed the in vivo potential of our probe in a subcutaneous xenograft mouse model. Fluorescence images were generated at multiple days after intravenous administration of 3 different doses of anti-GD2-IRDye800CW. Tumors were visualised with a clinical imaging device that is commonly used for FGS in patients and a preclinical system used for in vivo small animal imaging for fluorescence quantification (Fig. 1d and Supplementary Fig. S1 online). Fluorescence quantification showed a higher tumor-to-background ratio (TBR) for subcutaneous tumors of mice receiving a dose of 1 nmol and 0.3 nmol anti-GD2-IRDye800CW compared to 3 nmol (Fig. 1e), with a mean fluorescence intensity (MFI) of the tumor significantly higher for 1 nmol dose compared to 0.3 nmol (Fig. 1f). Based on the TBR and MFI curves, we defined an optimal time window for imaging 4 days after administration of 1 nmol anti-GD2-IRDye800CW, with a TBR of 5.2 (SEM ± 1.3) and MFI of 0.28 (SEM ± 0.1), comparable to preclinical TBR and MFI values previously reported for FGS agents successfully used in adult oncology^17–19^. Mice receiving the control antibody anti-CD52-IRDye800CW had a significantly lower TBR and MFI, compared to the 1 nmol dose (Fig. 1e, f, Supplementary Fig. S1 online), further demonstrating specific binding of anti-GD2-IRDye800CW to GD2 within tumors. No difference in fluorescence signal was found between tumors of different size (Supplementary Fig. S2 online and Supplementary Table S1 online).

### Orthotopic tumor engraftment demonstrates feasibility for fluorescence-guided surgery

**Figure 2:**
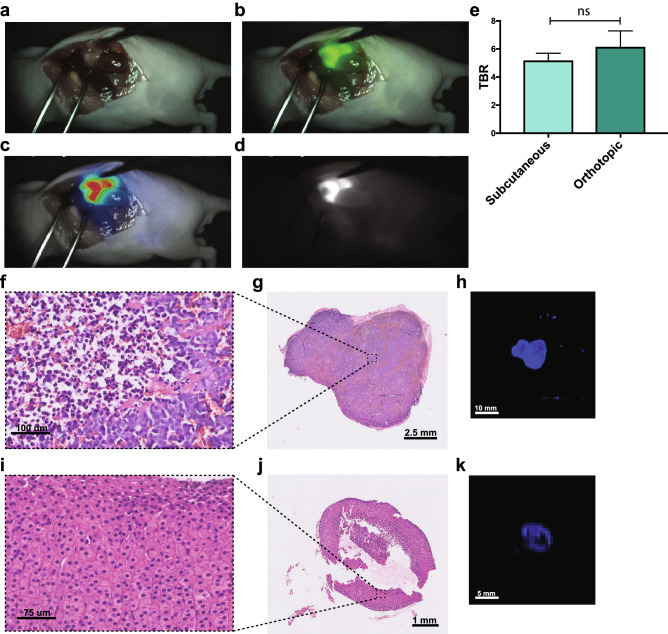
Orthotopic KCNR-derived tumor model confirms clinical potential of anti-GD2-IRDye800CW for FGS. (**a**–**d**) Representative images of FGS of an orthotopic KCNR-derived tumor 4 days post-injection using the Quest Artemis imaging system as a real time-navigator. (**a**) shows the white light view of the surgeon (**b**) the overlay with the fluorescence signal in green, (**c**) the overlay of the heatmap and (**d**) the fluorescence signal. (**e**) TBR obtained with the Pearl imaging system and compared to TBR from the subcutaneous model. Mean + SEM, non-significant (ns) *p* = 0.264. (**f**–**h**) Representative H&E images of a tumor section imaged at 40 × magnification. (**f**) depicts an enlargement from (**g**). (**h**) Representative 2D image of the same section imaged in the Odyssey Clx for fluorescence intensity. (**i**–**k**) Similar representative images as in (**f**–**h**) for the adrenal gland of a control mouse not engrafted with KCNR cells. n = 2 independent experiments with n = 5 mice per group.

**Figure 3:**
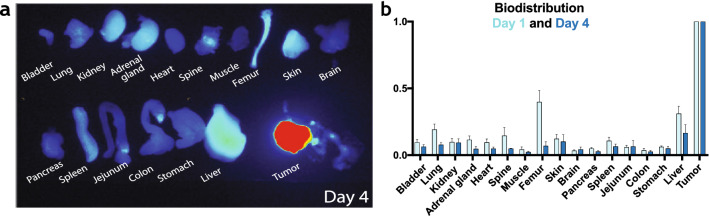
Biodistribution demonstrates the mostly hepatic clearance of anti-GD2-IRDye800CW. (**a**) Representative images at day 4 for the biodistribution of anti-GD2-IRDye800CW in multiple organs of orthotopic tumor-bearing mice receiving a 1 nmol dose imaged with the Pearl imaging system. (**b**) accumulative data at day 1 and 4. Mean MFI normalized to tumor + SEM. n = 2 independent experiments with n = 3–4 mice.

To investigate our tracer in a more clinical setting, we implemented an orthotopic model with KCNR cells transplanted in the adrenal gland, the most common location for NB^20^. Following the optimized conditions determined in the subcutaneous model, mice were intravenously injected with 1 nmol anti-GD2-IRDye800CW and the tumors were resected 4 days post injection guided by the Quest camera (Fig. 2a–d, Supplementary Video S1 online). In this surgical set-up, we defined a TBR of 6.1 (SEM ± 2.2), similar to the subcutaneous model (Fig. 2e) and detected no remaining fluorescent tissue after surgery (Supplementary Video S1 online). After harvesting the tumors, histology confirmed that NB cells on hematoxylin and eosin (H&E) staining overlapped with fluorescence of anti-GD2 (Fig. 2f–h), and, importantly, healthy adrenal gland from non-transplanted control mice only showed background levels of anti-GD2-IRDye800CW fluorescence (Fig. 2i–k). By quantifying the biodistribution, fluorescence in non-tumor tissue was only seen in the femur, as well as in the liver at day 1, which importantly was lower compared to tumor tissue and further diminished by day 4 (Fig. 3a, b), confirming the mostly hepatic clearance of anti-GD2-IRDye800CW. Overall, these data show that anti-GD2-IRDye800CW is a suitable probe for fluorescence tumor detection in vivo.

### Upfront dinutuximab-beta immunotherapy does not interfere with tumor detection

**Figure 4:**
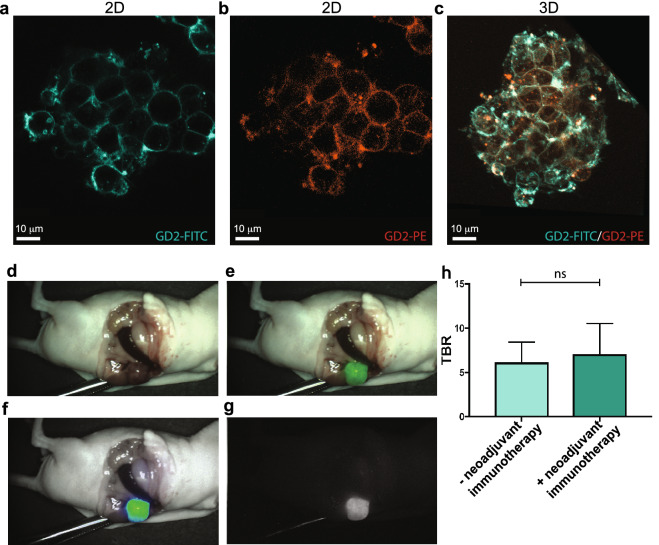
Anti-GD2 dinutuximab-beta treatment does not interfere with anti-GD2-IRDye800CW fluorescence. (**a**–**c**) Representative confocal images of KCNR cell 3D spheroids after incubation with anti-GD2-FITC (in cyan) (**a**), followed by anti-GD2-PE (in red) (**b**), or an overlay of both channels in 3D (**c**). n = 3 independent experiments. (**d**–**g**) Representative images of FGS in orthotopic NB tumor-bearing mice receiving upfront anti-GD2 immunotherapy, using the Quest Artemis imaging system as a real time-navigator. (**d**) shows the white light view of the surgeon, (**e**) the overlay with the fluorescence signal in green, (**f**) the overlay of the heatmap and (**g**) the fluorescence signal. (**h**) TBR measured from images recorded using the Pearl imaging system in mice receiving upfront anti-GD2 immunotherapy compared to mice without anti-GD2 upfront treatment, non-significant (ns) *p* = 0.503 (**d**–**h**) n = 2 independent experiments with n = 5 mice per group.

Currently, clinical trials are being initiated with neoadjuvant anti-GD2 immunotherapy prior to surgery in high-risk NB^21^. Therefore, we investigated whether this neoadjuvant immunotherapy would not interfere with the use of anti-GD2-IRDye800CW for FGS. In vitro we showed that 24 h after incubation with a human anti-GD2-FITC antibody (Fig. 4a), KCNR cells could effectively be stained a second time with a human anti-GD2 coupled to a different fluorophore; Phycoerythrin (PE) (Fig. 4b, c). This indicates that GD2 is not lost from the cell surface after antibody binding and that upfront anti-GD2 treatment should not interfere with membranal GD2 fluorescent imaging. To confirm this in vivo, mice with orthotopic induced tumors were pre-treated with a clinically derived dose of 1 nmol anti-GD2 for 2 cycles with 4 days in between, before receiving 1 nmol anti-GD2-IRDye800CW. In this model, the fluorescence signal in mice pre-treated with anti-GD2 dinutuximab-beta was sufficient for intraoperative fluorescence imaging and tumor resection (Fig. 4d–g) and TBRs (7.0 SEM ± 3.4) were comparable to those without pre-treatment (6.1 SEM ± 2.2) (Fig. 4h). This demonstrates that the use of neoadjuvant anti-GD2 immunotherapy is unlikely to diminish the fluorescence signal and that anti-GD2-IRDye800CW can be used for FGS in high-risk NB patients regardless of their order of treatment.

### Tissue microarray of different pathological tumor stages reveals consistent, yet heterogeneous GD2 expression in high-risk NB patients

**Figure 5:**
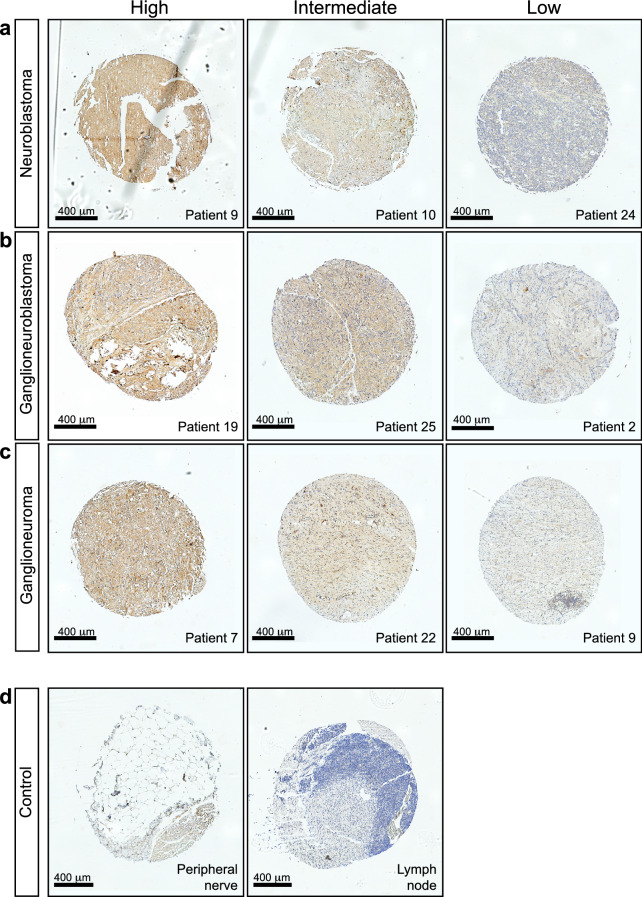
Tissue microarray of different stages of NB shows a heterogeneous expression of anti-GD2 between patients. (**a–d**) Representative images of anti-GD2 immunohistochemical staining on a tissue microarray (TMA) containing tissue of 28 patients in duplicate. (**a**) Three representative images of differential GD2 labelling (high, intermediate and low) for neuroblastoma tissues. (**b**) Three representative images of differential labelling for ganglioneuroblastoma tissues and (**c**) three representative images for ganglioneuroma tissues. (**d**) Anti-GD2 stainings on control peripheral nerve (left) and lymph node tissue (right).

To further investigate the extent of patients that can potentially benefit from anti-GD2-IRDye800CW guided surgery, we defined the expression of GD2 on a tissue microarray (TMA) consisting of tumor samples from 28 high-risk NB patients treated with chemotherapy (Supplementary Fig. S3 online). This demonstrated consistent expression of GD2 across multiple pathological tumor stages; neuroblastoma (Fig. 5a), ganglioneuroblastoma (Fig. 5b) and ganglioneuroma (Fig. 5c) and after chemotherapy, in line with previous literature^13,22^. Importantly, no signal was detected in control peripheral nerve and lymph node tissue (Fig. 5d). However, even when belonging to the same tumor category, heterogeneity between individual patients could be observed (Supplementary Fig. S3 online) and in each subtype we could identify samples with high, intermediate, or low GD2 expression (Fig. 5a–c).

### Patient-derived organoid lines demonstrate adequate tumor detection across differential GD2 expression levels

**Figure 6:**
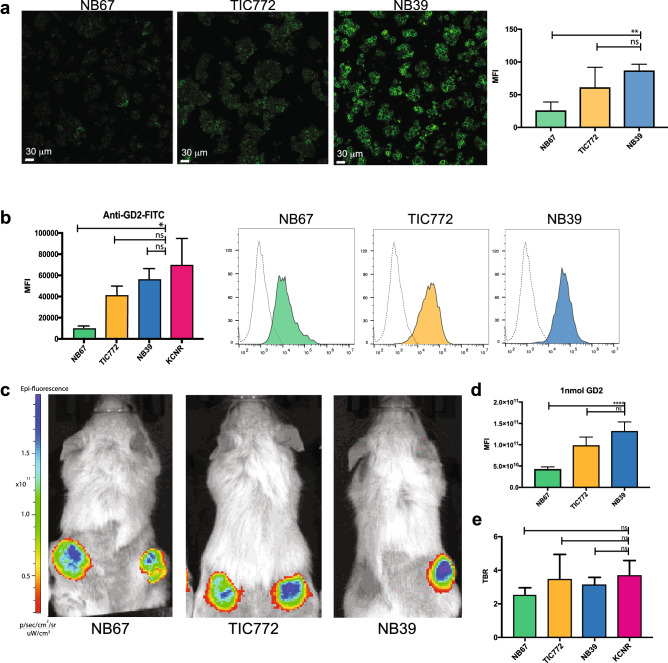
Patient-derived organoid lines representing heterogeneous GD-2 expression demonstrate adequate florescent signal in case of low GD2 expression*.* (**a**) Representative confocal images of the three different patient-derived NB organoid lines NB67, TIC772 and NB39 showing anti-GD2 staining at different intensities. Graphs (right panel) depict mean intensities + SEM, **p = 0.0028 for comparison of NB67 and NB39 and non-significant (ns) (*p* = 0.2365) for comparison TIC772 and NB39. (**b**) Accumulative data (left panel) and representative histograms (right panel) of anti-GD2-IRDye800CW staining analysed by flow cytometry. MFI + SEM *p = 0.05 for comparison of NB67 and KCNR, and non-significant (ns) for comparison TIC772 to KCNR (*p* = 0.3429) and NB39 to KCNR (*p* = 0.4857). (**c**) Representative in vivo images from patient-derived organoid xenograft models. (**d**) MFI for all concentrations in arbitrary units. Mean ± SEM. ****p < 0.001 for comparison of 1 nmol dose anti-GD2-IRDye800CW of NB67 to NB39 and non-significant (ns) for comparison of NB67 to NB39 *p* = 0.06 (**e**) TBR at day 4 of mice receiving anti-GD2-IRDye800CW. The TBRs of all organoid lines were non-significant (ns) when compared to the TBR of the KCNR cell line (*p* = 0.07 for comparing KCNR to NB67, *p* = 0.257 when comparing KCNR to NB39 and *p* > 0.999 when comparing TIC772 to KCNR).

In the last decade, organoid technology has become a valuable in vitro tool to study human cancer in a patient-specific manner^23^. Since the above results show that GD2 is not uniformly highly expressed in NB patients, we made use of three patient-derived neuroblastoma organoid lines; TIC772^24^, NB67 and NB39 (Supplementary Table S2 and unpublished data) that reflect the high, intermediate and low GD2 expression levels observed in patients, as shown by confocal imaging (Fig. 6a) and flow cytometry (Fig. 6b). Upon subcutaneous transplantation in vivo, tumors derived from each line showed detectable real time fluorescence with differential MFI as observed in vitro (Fig. 6c, d) but, importantly, no difference in TBR compared to KCNR-derived tumors (Fig. 6e). This indicates that even in patients with low GD2 expressing tumors, fluorescence obtained with anti-GD2-IRDye800CW will be sufficient for FGS.

## Discussion

By showing the feasibility of intraoperative fluorescence imaging of NB with anti-GD2-IRDye800CW, our preclinical study presents the first suitable molecular-targeted candidate for FGS to guide tumor resection in children. Although FGS with tumor surface antigen specific probes is increasingly implemented in adult oncology^11,25^, its application in pediatric oncology is still scarce. To the best of our knowledge, we here for the first time show that xenograft tumors derived from pediatric tumor cell lines or patient-derived organoids can be detected effectively in vivo using a real time intraoperative imaging system and thereby provide the first step towards closing this translational gap. Importantly, by using a clinical approved antibody combined with a NIR fluorescent probe, we are on a fast track for getting this tumor-specific tracer in a first in-child clinical trial.

Before testing efficacy in Phase II/III clinical trials, Phase I clinical trials will be required to determine the safety of anti-GD2-IRDye800CW. However, the immunotherapeutic antibody dinutuximab-beta, one component of our probe, has already been FDA approved. Based on our optimal dose of 1 nmol (0.15 mg) in mice, we expect an effective dose of 0.9 mg/kg (22 mg/m^2^) in children^26^, close to the dose of 20 mg/m^2^ that has been reported to have minimal toxicity in children^27^. In addition, the second part of our probe, the near-infrared dye 800CW has shown to be non- immunogenic in previous preclinical studies^28^ and has been safely administrated in multiple clinical trials^29,30^. One important limitation of our preclinical evaluation is that we cannot evaluate binding to GD2 potentially expressed on healthy tissue, because dinutuximab-beta is not cross-reactive with mouse GD2. However, other clinical studies have shown that GD2 expression is restricted to neurons, skin melanocytes, and peripheral sensory nerve fibers^31^ with expected intensity signals not-detectable compared to NB. In line with this, we did not obtain positive GD2 staining on control peripheral nerve and lymph node tissue in our TMA. Although safety levels of the conjugated probe still need to be carefully evaluated, these results are highly promising towards the outcome of such evaluations.

Timely progress into clinical trials might benefit children suffering from high-risk NB in multiple ways. Due to the localization of most NB tumors, their encasement in important vasculature and the surgeon’s challenge to discern tumor from healthy tissue, resection is almost never complete^4,32^. While standard of care, this also complicates understanding the long-term patient benefit of tumor resection. Indeed, although some studies report that gross total resection improves overall survival^7,33^ or event-free survival^34^, others claim no obvious survival benefit^35^. Bias in determining the extent to which tumor resection was complete might contribute to this discrepancy. This is now based on the subjective impression of the surgeon, which is known to have a poor correlation with the results of post-operative imaging^34^. Introduction of FGS for NB resection, will provide an additional modality to quantify tumor cells or tissue remaining, based on fluorescence signal. The extent of resection can thereby be more accurately determined, which opens up new possibilities to reliably assess the effect of surgery on overall survival. In addition, a more accurate quantification of remaining tumor tissue, will help guide decision making on post-surgery course of treatment, and potential benefit of dinutuximab-beta immunotherapy in particular. Most importantly, by providing a precise surgery tool to resect tumoral tissue with higher confidence, chances of relapse due to remaining tumor might decrease. At the same time, damage to surrounding healthy organs and vasculature can be prevented, thereby lowering the risk of surgical complications that is still high in NB patients undergoing surgery^5^.

Considering the overexpression of GD2 on neuroblastoma cells^13^, we expect our GD2 specific tracer to be of clinical value for the majority of NB patients, which was confirmed by our TMA results showing GD2 expression on NB tissue in the majority of patients, across tumor subtypes and after chemotherapy treatment. Importantly, although we observed heterogeneous expression of GD2, with some patients only expressing intermediate or even low expression levels, experiments with patient-derived organoid lines reflecting these expression levels, showed that TBRs of subsequent xenografted tumors were still sufficiently high to discriminate tumor from healthy tissue, even for low expressing organoid lines. Finally, anti-GD2 is increasingly used for immunotherapy in high-risk NB^36^. Currently, after surgery, but ongoing trials will assess the advantage of neoadjuvant treatment^37,38^. Therefore, we also confirmed that anti-GD2-IRDye800CW can still detect NB tissue after upfront anti-GD2 treatment, further validating the wide range of patients that could potentially benefit from anti-GD2-IRDye800CW guided surgery.

In conclusion, we here present a first pediatric cancer specific tracer with potential for the vast majority of high-risk NB patients to guide tumor resection with greater accuracy, thereby lowering the risk of surgical complications and reducing the incidence of relapse. In addition, we envision the comprehensive preclinical evaluation pipeline presented, using both patient derived cell line and organoid xenograft models and encompassing multiple imaging technologies, to be highly applicable for the development of other targeted probes.

## Methods

### Antibody conjugation

Chimeric monoclonal antibody Dinutuximab-beta (Qarziba, USA) was conjugated to the NIR fluorophore IRDye800CW, (LI-COR Biosciences, Nebraska, USA), as previously described^19^. A degree of labelling (DoL) between 1.0 and 1.5 was considered successful. As a control, the antibody alemtuzumab directed against CD52 present on the surface of mature lymphocytes, was also conjugated to IRDye800CW.

### Human cancer cell lines

After evaluation of different neuroblastoma cell lines for GD2 expression, the neuroblastoma cell line SMS-KCNR (KCNR)^16^ was selected based on GD2 expression and in vitro and in vivo growth rate. The human colon colorectal cancer cell line HT-29 was used as a negative control. KCNR line was cultured in Dulbecco’s modified Eagle’s medium (DMEM)-GlumaMAX containing low glucose, 10% Fetal Bovine Serum (FBS), 1%ml NEAA, 100 IU/ml penicillin and 100 ug/ml streptomycin. HT-29 cells were cultured in RPMI-1640 medium (Life technologies, Gibco) with 10% FBS and 100 IU/ml penicillin. Both lines were cultured in a humidified incubator at 37 °C and 5% CO_2_ and free of *Mycoplasma* species.

### Patient-derived neuroblastoma organoids

Patient-derived neuroblastoma organoids^20,24^ (Kholosy et al. submitted) were grown in Dulbecco’s modified Eagle’s medium (DMEM)-GlumaMAX containing low glucose with addition of 20% Ham’s F-12 Nutrient Mixture, B-27 Supplement minus vitamin A, N-2 supplement, 100 IU/ml penicillin, 100 ug/ml streptomycin, 20 ng/ml epidermal growth factor (EGF), 40 ng/ml fibroblast growth factor-basic (FGF-2), 200 ng/ml insulin-like growth factor (IGF-1), 10 ng/ml platelet-derived growth factor AA (PDGF-AA) and 10 ng/ml platelet-derived growth factor BB (PDGF-BB).

### Flow cytometry

KCNR and HT-29 were grown to 90% confluency and detached with TrypleLE. Organoids were processed into single cells using 200 ul accutase. Cells were adjusted to 0.5 × 10^6^ viable cells per tube in FACS buffer and incubated with 200 μl phosphate-buffered saline (PBS) and 2 μl anti-GD2-IRDye800CW, or anti-CD52-800CW, as a negative control. Organoids were incubated with 2 μl anti-GD2-FITC (mouse 14g2a, Biolegend). After incubation, cells were washed three times in ice-cold PBS and resuspended in 500 μl PBS containing propidium iodine (PI) to stain dead cells. Samples were acquired on a LSRII flow cytometer (BD Biosciences, Singapore) and analysis was performed using FlowJo software (TreeStar, Ashland, Oregon, United States, version 10.6.2).

### 3D confocal imaging

KCNR cells were transferred to a 96 well sensoplate microplate (Greiner BIO-ONE) 24 h prior to imaging, allowing the cells to form 3D spheroids, due to the low adherence conditions, and incubated with anti-GD2-FITC (mouse 14g2a, Biolegend, 1/200) overnight at 4 °C. The following day, the cells were washed 3 times with medium before incubation with anti-GD2-PE (mouse 14g2a, Biolegend, 1/200) for 15 min on ice.

Patient-derived organoids were incubated directly after culture for 30 min with anti-GD2-FITC on ice before imaging. Imaging was performed on a confocal microscope using a 25X 0.8 NA objective (SP8 Leica microscope, LSM880 Zeiss microscope). 3D rendering was performed using Imaris (Bitplane).

### Ethics

All human organoid samples were obtained from the biobank of the Princess Màxima Center (PMCLAB2019.037). Authorizations were obtained from the medical ethical committee of UMC Utrecht (METC UMCU) to ensure compliance with the Dutch Medical Research Involving Human Subjects Act, and informed consent was obtained from all donors. All animal experiments were approved by the Animal Welfare Committee of either the LUMC and the Princess Máxima Center and carried out in compliance with both local and international regulations.

### Generation of neuroblastoma xenograft models

#### Mice

Six-week-old athymic nude female mice (CD1-Foxn1^nu^, Charles River Laboratories) were used for xenografting of KCNR cells and NSG-mice (bred in house) for organoid xenograft models.

#### Subcutaneous xenograft models

On average, 1.0 × 10^6^ KCNR cells or 1.0 × 10^6^ single cells from patient-derived organoids were injected subcutaneously at 2–4 dorsal sites in 50 μl 50% medium/50% BME. Throughout the injection of tumor cells and imaging procedures, animals were anesthetized with 2.5% isoflurane for induction of anesthesia and 2% isoflurane for maintenance with a flow of 0.5 l/min.

#### Orthotopic xenograft model

Mice were injected with 1:1:2 Hypnorm/Dormicum/PBS for analgesia. After 30 min, mice were anesthesized with 3% isoflurane and positioned with the left flank facing upward. After opening the skin and incision of the retroperitoneum, 1.0 × 10^6^ KCNR cells were injected in 10 μl medium in the left adrenal gland using a 0.5–10 μl syringe. Tumor growth was followed up by palpation of the back three times per week. When tumors were approximately 8 × 8 mm, mice were injected with anti-GD2-IRDye800CW and imaging performed. In experiments investigating the effect of neoadjuvant dinutuximab-beta treatment, mice were pretreated with 1 nmol in 50 µl PBS 3 weeks after engraftment, followed by a second dose 4 days later. 8 days after the first dose, 1 nmol of anti-GD2-IRDye800CW was administered for subsequent imaging.

### In vivo imaging of FGS probes

Mice bearing subcutaneous tumors starting from a size of 8 × 8 mm were intravenously injected in the tail vein with 0.3 nmol, 1 nmol or 3 nmol anti-GD2-IRDye800CW in 50 µl PBS. Fluorescence signal was measured using both the Pearl Trilogy Small Animal imaging system (LI-COR Biosciences, Lincoln, Nebraska, USA) and the Quest Artemis imaging system (Quest Medical Imaging, Middenmeer, The Netherlands). When the mice had multiple tumors, a size of 5 × 5 mm was considered the lower threshold to be included for analysis. Mice bearing organoid-derived subcutaneous tumors, were imaged by the IVIS Spectrum In Vivo Imaging System (Perkin Elmer, Waltham, MA, USA). Control mice were injected with antibody CD52-IRDye800CW (1 nmol). For orthotopic KCNR and patient derived organoid xenograft models, mice were injected with the optimal dose of 1 nmol anti-GD2-IRDye800CW.

#### TBR calculation and measurement of biodistribution

Regions of interest were drawn based on the visible tumor to measure the MFI on the Pearl and the IVIS. Imaging data from the Pearl, Quest and IVIS were analysed using respectively Pearl Cam Software, ImageJ (W. Rasband, Bethessa, Maryland, USA) and Living Image Software (Perkin Elmer, Waltham, MA, USA, version 4.7.3.). TBRs were calculated by dividing the mean fluorescence intensity (MFI) of the tumor by the adjacent abdominal background signal in donut shape surrounding the tumor. Biodistribution of anti-GD2-IRDye800CW was determined by measurement of MFI from multiple organs at 24 and 96 h after administration in mice bearing subcutaneous tumors and injected with 1 nmol anti-GD2-IRDye800CW.

### Generation of patient tissue microarray

A tissue micro array (TMA) was created using paraffin-embedded tissue blocks of patients diagnosed with high-risk neuroblastoma, who underwent surgical resection after chemotherapy treatment between 2014 and 2017 at the Princess Máxima Center, Utrecht, The Netherlands. Three different TMA blocks were created containing the three different histological subtypes found in 28 different patients according to the International Neuroblastoma Pathology Classification (INPC); neuroblastoma (n = 18), ganglioneuroblastoma (n = 20) and ganglioneuroma (n = 20)^22^. Samples were obtained during debulking surgery, formalin fixed, confirmed to represent neuroblastoma tissue by a professor in pediatric oncology pathology and histologically scored as neuroblastoma, ganglioneuroblastoma or ganglioneuroma. Samples were placed on the TMA in duplicate. Control samples of healthy tissue from peripheral nerves and lymphoid tissue were added. The TMA was subsequently stained with anti-GD2 (mouse 14g2a, Biolegend, 1/50).

### Histological analysis

After euthanizing the mice, tumors were surgically removed and fixed in formalin. Tumors were sectioned and scanned on the Odyssey Clx (LI-COR Biosciences, Lincoln, NE, USA). A solid state laser diode tuned at 785 nm was used for optimal excitation of the fluorophore IRDye800CW and light was collected in the 800 nm channel for evaluation of the fluorescence location. Slides of subsequent sections were stained with haematoxylin–eosin.

### Statistical analyses

Statistical analysis was performed using Graphpad Prism software (version 7, GraphPad Software Inc, La Jolla, CA, USA). The Area Under the Curve (AUC) was calculated for the different dose groups and comparison of means were performed with the unpaired t-test. All other comparisons of means were performed with the Mann Whitney U test.

## Supplementary information

Supplementary Information.

Supplementary Video.

## Data Availability

All data is included in either the main manuscript or Supplementary Information.
