# Supplementary material for: Anti-GD2-IRDye800CW as a targeted probe for fluorescence-guided surgery in neuroblastoma
Source: Sci Rep. 2020 Oct 19;10:17667. doi: 10.1038/s41598-020-74464-4 (PMC7573590; doi:10.1038/s41598-020-74464-4)
Supplement: Supplementary file 1 — Supplementary Information. [file 41598_2020_74464_MOESM1_ESM.pdf]

Supplementary Information

Anti-GD2-IRDye800CW as a targeted probe for fluorescence-guided surgery in neuroblastoma

Lianne M. Wellens[1,2,3#], Marion M. Deken[4#], Cornelis F.M. Sier[4], Hannah R. Johnson[1,2,3], Fàtima de la Jara Ortiz[1], Shadhvi S. Bhairosingh[4], Ruben D. Houvast[4], Waleed M. Kholosy[1], Victor M. Baart[4], Annique M.M.J. Pieters[5], Ronald R. de Krijger[1], Jan J. Molenaar[1], Ellen J. Wehrens[1,2,3], Johanna F. Dekkers[1,2,3], Marc H.W.A. Wijnen[1], Alexander L. Vahrmeijer[4], Anne C. Rios[1,2,3*].

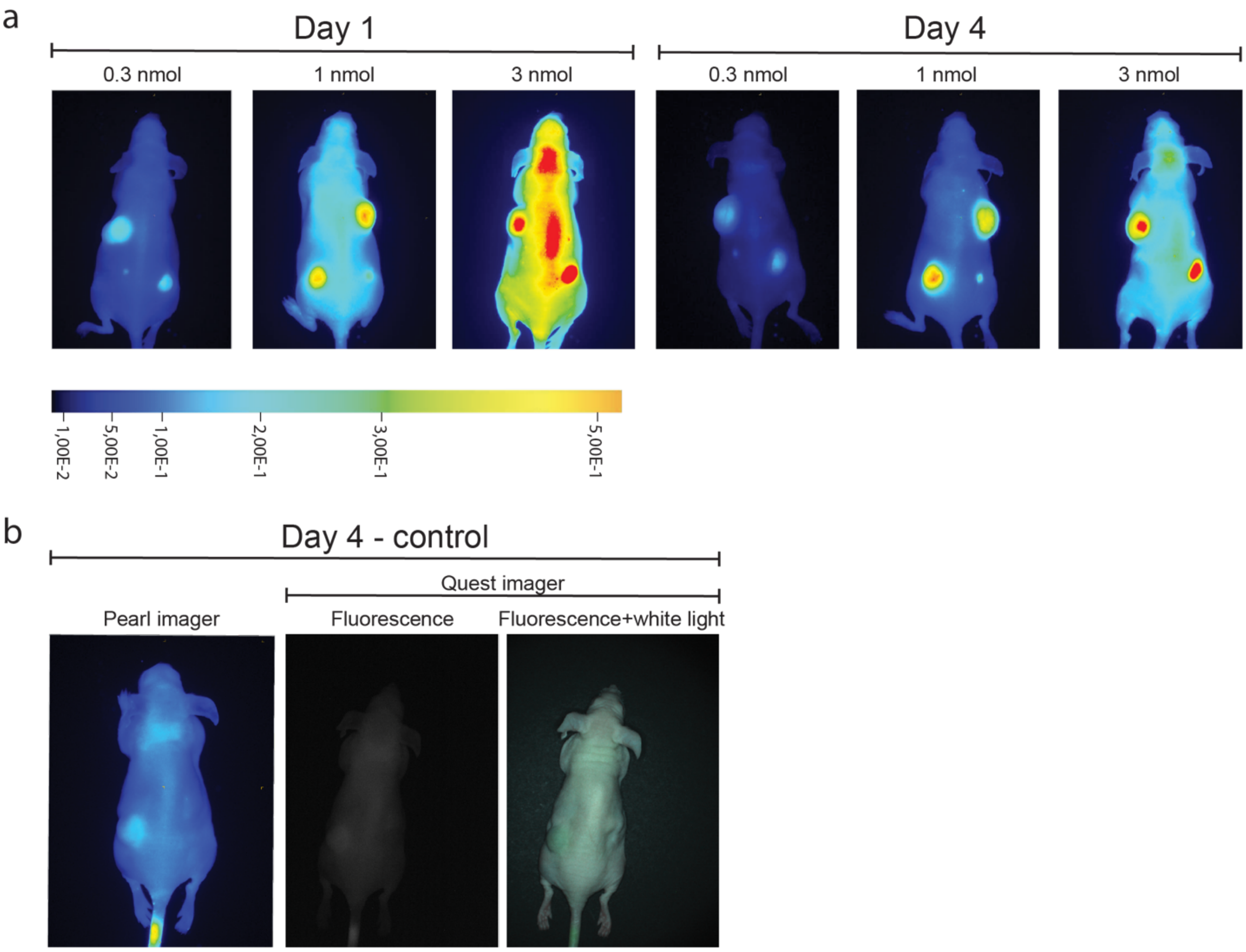

Supplementary Figure S1. *In vivo* imaging of anti-GD2-IRDye800CW and anti-CD52-IRDye800CW in subcutaneous xenograft KCNR-derived model.

a) Representative *in vivo* fluorescence images at day 1 (left panel) and day 4 (right panel) of the 3 different dose groups of anti-GD2-IRDye800CW acquired with a quantitative device (Pearl Small Animal In Vivo Imager) of mice bearing subcutaneous human KCNR-derived tumors. **b)** Representative images generated with the Pearl Small Animal In Vivo Imager (Left panel) and the Quest imager (middle and right panels) 4 days after administration of 1 nmol control anti-CD52-IRDye800CW in mice bearing subcutaneous KCNR-derived NB tumors.

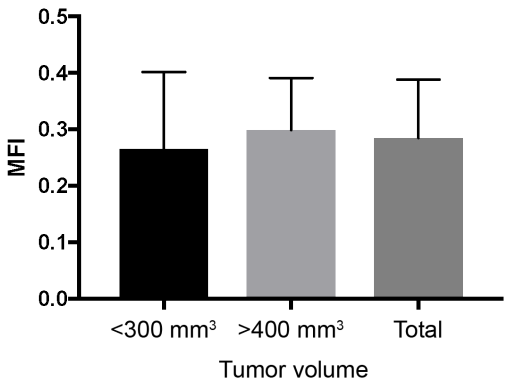

Supplementary Figure S2. Fluorescence of anti-GD2-IRDye800CW does not vary with tumor size. Graph showing MFI from subcutaneous tumors of different volumes at day 4 after injecting 1 nmol anti-GD2-IRDye800CW. Mean + SEM of n = 3-4.

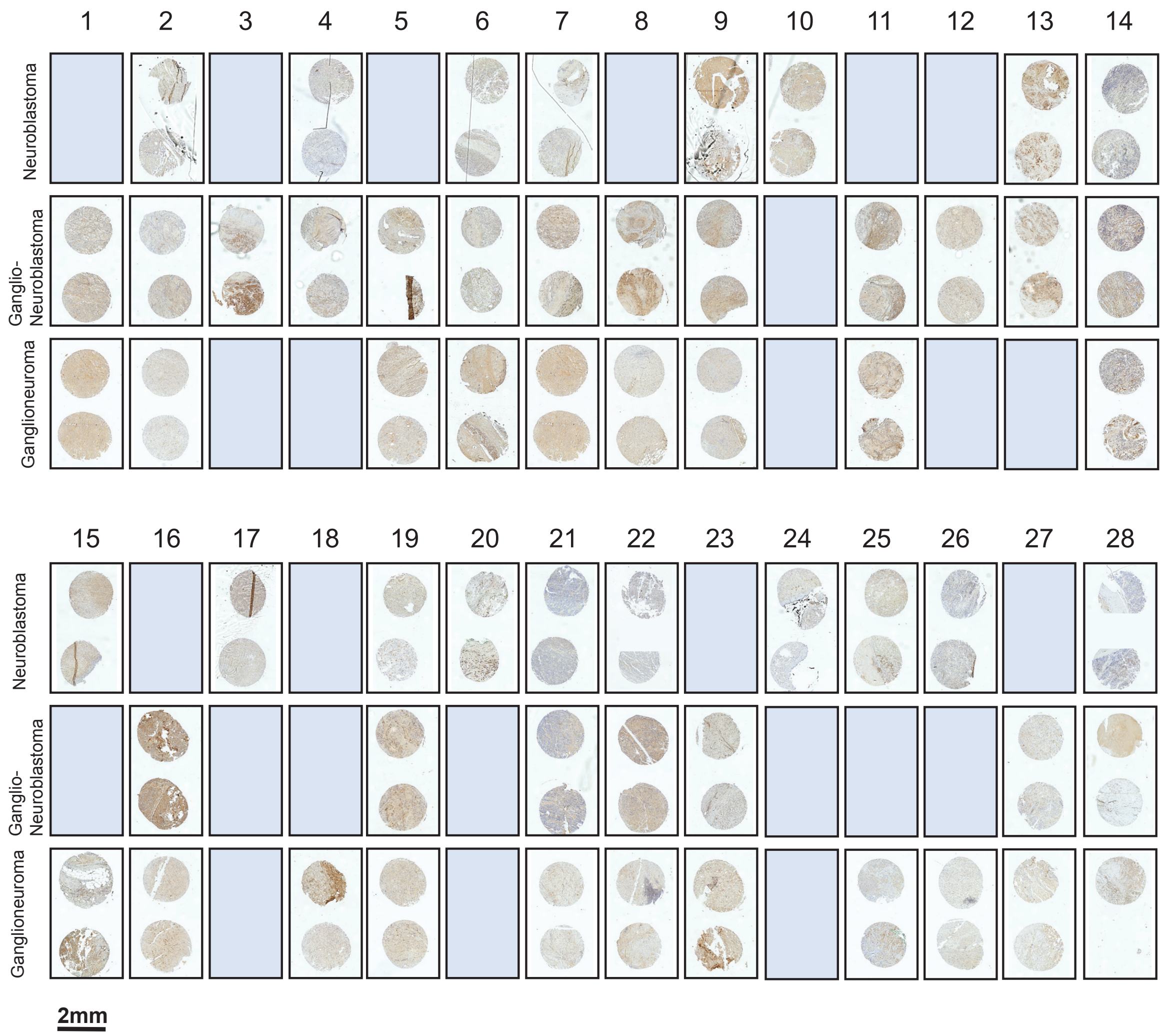

Supplementary Figure S3. Overview of the different NB samples used for the TMA. Representative microscopy images demonstrating the expression of GD2 across multiple pathologic stages (neuroblastoma, ganglioneuroblastoma and ganglioneuroma) in tumor tissue from 28 high-risk NB patients after treatment with chemotherapy.

Subcutaneous tumors					
**mouse**	tumor size (lxh)	tumor size (mm^3)	MFI tumor	MFI background	TBR
**R-72**	11x9	445,5	0,242	0,0584	4,14383562
**R-72**	6x8	144	0,304	0,0541	5,61922366
**LL-73**	10x9	405	0,214	0,0471	4,54352442
**LL-73**	8x7	196	0,115	0,0298	3,8590604
**LL - 64**	10x9	405	0,419	0,0526	7,96577947
**LL - 64**	10x11	550	0,321	0,0629	5,10333863
**O**	8x8	256	0,378	0,075	5,04

Supplementary Table S1. Fluorescent detection related to tumor size.

Tumor dimensions (size and volume) and corresponding Pearl Small Animal In Vivo Imager results (MFI and TBR) of the subcutaneous tumors included in Supplementary Figure S2.

Organoid line	Age at diagnosis (months)	INSS stage	Type of Tissue	Location	PA	MYCN amplified	Mutations	Culture conditions
**NB 39**	24	4	Biopsy relapse	Lymph node	Poorly differentiated NB	Yes	Unknown	Organoid Medium
**NB 67**	29	4	Biopsy primary tumor	Adrenal gland	Poorly differentiated NB	Yes	ALK	Organoid Medium
**TIC 772**	36	4	Biopsy primary tumor	Adrenal gland	Poorly differentiated NB	No	No	TIC Medium

Supplementary Table S2. Pathological and molecular profile of patient-derived neuroblastoma organoid lines. Characteristics of patient NB tissue from which organoid lines were generated. NB = Neuroblastoma, INSS = The International Neuroblastoma Staging System, PA = pathology, MYCN = MYCN-gene, ALK = Anaplastic Lymphoma Kinase gene.

Supplementary Video S1

Representative video of a surgical procedure performed on 5 mice in total bearing an orthotopic neuroblastoma tumor. Video was recorded on the Quest Artemis Imaging system, at 50 ms excitation in the 800 channel. Anti-GD2-IRDye800CW was administered 4 days before surgery. The left upper panel shows the normal vision of the surgeon with white light, the right upper panel shows the overlay of the fluorescence and white light with the tumor labelled in green. The left lower panel shows the overlay of the white light and the fluorescence heatmap and the right lower panel shows the fluorescence signal captured by the 800 filter on the Quest.
